# Effect of Free Volume on Curcumin Release from Various Polymer-Based Composite Films Analyzed Using Positron Annihilation Lifetime Spectroscopy

**DOI:** 10.3390/ma14195679

**Published:** 2021-09-29

**Authors:** Jong-Whan Rhim, Saygin Kuzeci, Swarup Roy, Necmettin Akti, Cumali Tav, Ugur Yahsi

**Affiliations:** 1 Department of Food and Nutrition, BioNanocomposite Research Institute, Kyung Hee University, 26 Kyungheedae-ro, Dongdaemun-gu, Seoul 02447, Korea; swaruproy2013@gmail.com or; 2 Physics Department, Faculty of Arts and Sciences, Marmara University, Kadikoy, Istanbul 34722, Turkey; drsaygin@uokirkuk.edu.iq (S.K.); necm23@yandex.com (N.A.); ctav@marmara.edu.tr (C.T.); 3 Physics Department, College of Education for Pure Science, Kirkuk University, Kirkuk 36001, Iraq

**Keywords:** positron annihilation lifetime spectroscopy (PALS), free volume, curcumin, polymers, composite film, release

## Abstract

This work reports the effects of free volume on curcumin release in various polymer-based composite films. Curcumin-reinforced biocomposite films were fabricated with natural biopolymers (carrageenan and chitosan) and bioplastics (poly(lactide) (PLA) and poly (butylene adipate-co-terephthalate) (PBAT)) via the solvent casting method. The curcumin release test was performed using an aqueous medium, and it was found that it was released the fastest in the carrageenan film, followed by the chitosan, PLA, and PBAT films, presumably owing to the dissimilarity of the polymer matrix. The free volume of the polymer films was determined using positron annihilation lifetime spectroscopy (PALS) to understand the release phenomena of curcumin. The free volume fraction was varied and reliant on the type of polymer, with the highest in the PBAT-based film followed by the PLA-, chitosan-, and carrageenan-based films. The free volume method helps analyze the release of bioactive compounds in a polymer matrix and may help to achieve a better understanding of the release of bioactive compounds.

## 1. Introduction

In recent years, the manufacture of eco-friendly functional packaging films integrating numerous natural functional materials has garnered significant attention in the active packaging field [1,2]. For the production of eco-friendly packaging films, natural biopolymers such as polysaccharides, proteins, blends, and various bioplastics such as PLA (poly(lactide)), PBAT (poly(butylene adipate-co-terephthalate)), and PHAs (polyhydroxyalkanoates) are combined with active functional materials, such as natural plant extracts and functional nanomaterials [1,3,4]. In this context, curcumin could be a good choice as a naturally produced bioactive functional material. Recently, curcumin has been used to manufacture bioactive packaging, and in biomedical applications. Some of the results showed it could be a promising candidate for manufacturing bioactive packaging films [5,6,7,8,9,10,11]. The functional material curcumin is released from the film upon contact with food to provide functionality through dissolution, diffusion, partitioning, swelling, and targeting. The release of active functional components from packaging films is commonly evaluated using various food simulation solutions. It is known that the release of bioactive compounds from packaging films to food simulants depends on the polymer matrix [12]. The change in release with the polymer matrix may be due to differences in the swelling rate (polymer), the solubility (active compound), and the integrity (polymer) [13].

To further understand the phenomena of the release of bioactive molecules, one of the relatively lesser known methods, i.e., measurement of the free volume of polymer films, can be used. Open spaces called “free volume” exist in the polymer structure as the chemical form changes. The free volume model of a polymer can explain several critical physical phenomena such as viscosity [14,15,16,17,18,19,20,21], ionic conductivity [22,23], relaxation [24], glass transition temperature, and other mechanical and thermodynamic properties. Several techniques such as X-ray or neutron scattering, differential scanning calorimetry, electron microscopy, dynamic mechanical study, and Fourier transform infrared spectroscopy can be utilized to measure the free volume of a polymer and to indirectly provide knowledge of the free volume of polymers and their composites [25]. One of the direct methods of determining the free volume is positron annihilation lifetime spectroscopy (PALS), which is commonly utilized to measure the size, density, and size distribution of the free volume in a polymer matrix [26,27].

In materials, positrons localize in open spaces between molecules away from the positive charges of nuclei. In an open space, if a positron interacts with an electron, bound-state positronium (Ps) may be formed in two states: one with a single state (singlet) called para-positronium (p-Ps), and the other with a triple state (triplet) called ortho-positronium (o-Ps). In para-positronium (p-Ps), the electron and the positron have anti-parallel spins, having a lifetime of 125 ps in a vacuum and appreciably the same lifetime in matter. However, in the ortho-positronium (o-Ps) states, the electron and the positron have parallel spins, having a lifetime of 132 ps in a vacuum and a drastic change to 0.5–5 ns in matter. Therefore, we are interested in the latter to relate the lifetime to the free volume as the open space. The size and concentration of the free volume are extracted from the lifetime spectrum in terms of the ortho-positronium (o-Ps) lifetime (τ_3_) and intensity (*I_3_*) using the PALS system, a novel tool to investigate holes [28].

Details about the free volume of an active packaging film can help understand the release of the active compounds from the film. The main aim of the present work was to estimate the free volume of functional packaging films prepared with two polysaccharides (carrageenan and chitosan) and two bioplastics (PLA and PBAT) incorporated with a biofunctional compound, curcumin. The release profile of curcumin from the functional films was investigated. The free volume of the functional films was measured over a temperature range of 10 to 80 °C using PALS. The results of free volume analysis were correlated with the release of curcumin from the functional films.

## 2. Materials and Methods

### 2.1. Materials

Food-grade κ-carrageenan and chitosan (CS-001, viscosity: 110 cp in 1% acetic acid solution at 25 °C; deacetylation: 90%) were obtained from Hankook Carragen (Whasoon, Jeonnam, Korea) and Samsung Chitopia (Seoul, Korea), respectively. Curcumin was obtained from Sigma-Aldrich (St. Louis, MO, USA). Glycerol and Tween 80 were procured from Daejung Chemicals & Metals Co., Ltd. (Siheung, Gyeonggi-do, South Korea). PLA (PLLA, Biomer^®^ L9000; average molecular weight: 200 kDa) was bought from Biomer Inc. (Krailling, Germany), and PBAT (EnPol PBG7070, m.p.: 125 °C; specific gravity: 1.20–1.25) was acquired from S-EnPol Co. Ltd., Wonju, Korea.

### 2.2. Preparation of Curcumin-Added Films

#### 2.2.1. Natural Biopolymer-Based Films

The carrageenan- and chitosan-based films were fabricated using the solution casting process [8]. For solution preparation of the carrageenan-based functional film, 0.04 g curcumin (1 wt % of the polymer) was first added to 150 mL of distilled water containing 1 wt % of Tween 80 as an emulsifier while vigorously stirring. Subsequently, 30 wt % of glycerol was mixed, and 4 g of carrageenan was slowly mixed and heated (90 °C for 30 min) with constant agitation. A chitosan-based functional film solution was prepared following the same process using 1% acetic acid solution. The film solutions were cast on Teflon film-coated glass plates (24 × 30 cm^2^) and dried at room temperature for 48 h. The dried films were peeled off the plates and conditioned in a humidity chamber (25 °C, 50% RH) for two days before further testing.

#### 2.2.2. Bioplastic-Based Films

The PLA/curcumin and PBAT/curcumin films were also fabricated using the solution casting method [9,10]. First, 0.04 g of curcumin was added to 100 mL of chloroform and dispersed for 2 h with gentle agitation, and then 4 g of each bioplastic (PLA or PBAT) was gradually integrated into the curcumin solution and dissolved (48 h with gentle stirring). The film-making solutions were cast evenly on Teflon film-coated glass plates and dried at room temperature for 48 h.

Additional carrageen/curcumin, chitosan/curcumin, PLA/curcumin, and PBAT/curcumin film sheets with a thickness of about 2 mm for the free volume analysis were prepared using the same procedure. In addition, the control carrageenan, chitosan, PLA, and PBAT film sheets were prepared using the same method without adding curcumin.

### 2.3. Curcumin Release Test

The quantity of curcumin released from the curcumin-containing functional films to water was measured following Roy and Rhim [8]. The functional film sample (2.5 × 2.5 cm^2^) was added to 20 mL of distilled water and then gently shaken at 37 °C. Curcumin release tests were performed on the natural biopolymer-based films and bioplastic-based films for 6 h and 96 h, respectively. An amount of 3 mL of the extracted sample solution was taken at predetermined time intervals, and the spectrophotometric absorbance was measured at 420 nm. The amount of curcumin was calculated using a standard curve and stated as μg curcumin/mm^2^ film.

### 2.4. Positron Annihilation Spectroscopy Analysis

Positron annihilation lifetime spectroscopy (PALS) was executed using a fast-fast coincidence system evaluating the time interval between the prompt γ-ray of 1274 keV as the start signal and the annihilation γ-ray of 511 keV as the stop signal. The positron source was prepared by placing a solution of about 30 μCi of ^22^NaCl on a thin aluminum foil (5 μm thick) inserted between two pieces of film samples with a thickness of 2 mm or more.

For γ-ray detection, a plastic scintillator (BC422, Saint-Gobain Crystals, Hiram, OH, USA) was connected to photomultiplier tubes (PMT R2059, Hamamatsu Photonics Deutschland GmbH, Herrsching, Germany) mounted on a PMT Base (265A, Ortec AMETEK GmbH, Meerbusch, Germany) operating at negative 2100 volts. There were two constant fractional differential discriminators for the window settings of 1274 keV and 511 keV, and timing signals (CFDD 583B, Ortec AMETEK GmbH, Meerbusch, Germany) were used. A time-to-amplitude converter (TAC 266, Ortec AMETEK GmbH, Meerbusch, Germany) converted pulses of different heights into a time-to-pulse-height signal. The converted signals were fed to a multi-channel analyzer (Ethernim MCA 919E, Ortec AMETEK GmbH, Meerbusch, Germany). The spectroscopic data obtained from MCA were analyzed using the LT polymer [29] to obtain the lifetime and intensity, providing facts on the free volume. The resolution of the system was measured using a Si crystal as a reference. The resolution was about 350 ps, and the source contribution was about 10.5%, with lifetime contributions of 0.2 ns and 0.4 ns and respective intensities of 80% and 20%. For good statistics, a million counts were taken for each run. All the other parameters are shown in Table 1 and Table 2. 

Typically, three types of lifetime data such as the para-positronium (p-Ps) lifetime (τ_1_) and intensity (*I*_1_), the direct annihilation lifetime (τ_2_) and intensity (*I*_2_), and the longest-lived component’s ortho-positronium (o-Ps) lifetime (τ_3_) and intensity (*I*_3_) can be obtained from analysis of the positron lifetime spectra using the LT polymer [28,29]. The LT code is a dedicated analysis program to fit PALS data using deconvolution of the lifetime spectrum into a few exponential components. The o-Ps lifetime can be used as a measure of the free volume size. Ps is considered to be localized in a spherical volume of radius *R*. The overlap integration of the Ps wave function has to be taken with the surrounding electron cloud layer of thickness *δR* = *R*_0_ − *R*. This parameter is chosen as an empirical parameter describing the penetration of the Ps wave function into the bulk inside the spherical potential well. The relationship between the radius *R* and *τ*_3_ allows for the evaluation of the mean free volume hole size with a spherical approximation proposed by the Tao–Eldrup model [30,31] as follows:

(1) $$\frac{1}{\tau_{3}\left(ns\right)}=2\left(1-\frac{R}{R_{0}}+\frac{1}{2\pi }\sin\frac{2\pi R}{R_{0}}\right)$$

with *δR* = 0.1656 nm [28,32]. The free volume hole size can then be calculated as follows:

(2) $$\upsilon_{f}\left(\tau_{3}\right)=4\pi R^{3}/3$$

The free volume fraction (*f_υ_*) can be linearly related to the intensity and free volume, as suggested by Kobayashi et al. [28,32,33]:

(3) $$f_{\upsilon }=AI_{3}\upsilon_{f}\left(\tau_{3}\right)$$

where *A* is a constant; the constant *A* heavily depends on material-related properties such as the positronium formation probability and the local electron density. Here, even if we did not have any exact information about the value of *A*, we employed 0.0018 Å^3^, presented in the literature [28], to at least show its trend as a representation of the free volume fraction.

## 3. Results and Discussion

### 3.1. Curcumin Release Test

**Figure 1:**
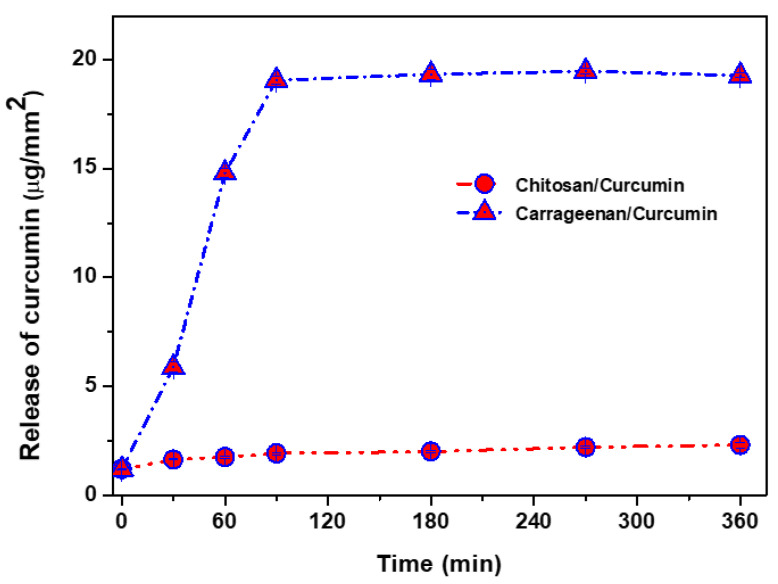
Release of curcumin from the natural biopolymer-based functional films.

**Figure 2:**
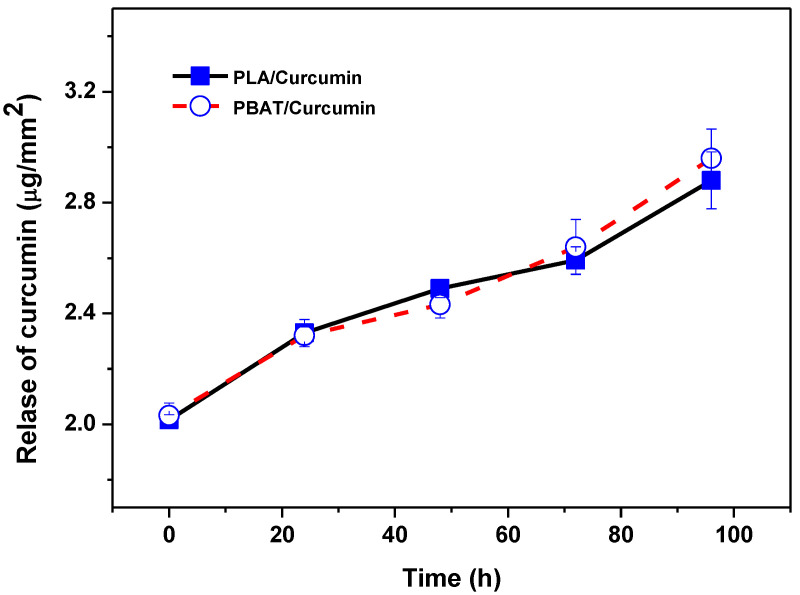
Release of curcumin from the bioplastic-based functional films.

Curcumin was consistently spread in all the polymers to form transparent yellowish flexible films, except for the translucent PBAT film [8,9,10]. The release patterns of curcumin from the natural biopolymer-based and bioplastic-based functional films are shown in Figure 1 and Figure 2, respectively. The release of curcumin was greatly reliant on the type of polymer matrix. In general, curcumin release was faster and greater with the natural biopolymer-based films than the bioplastic-based films. The curcumin release rate also varied, being reliant on the type of natural biopolymer. Figure 1 shows that curcumin release was much faster and greater from the carrageenan-based film than the chitosan-based film. Such a difference in the curcumin release rate is mainly due to the difference in the polymeric films’ water solubility and swelling ratio, leading to the different diffusion rates of curcumin [8]. However, there was no noteworthy variance in the release profile of curcumin in the bioplastic-based films (Figure 2), mainly attributed to the hydrophobicity of both the PLA- and PBAT-based films with low water solubility and a low swelling ratio [9,10]. In general, various factors such as the solubility and swelling ratio of the polymer matrix, the solubility and compatibility of the bioactive compounds, and the integrity of the composite polymer are known to play a vital role in releasing the active compounds [9,34,35]. In addition, using the free volume measured as a result of PALS, we tried to explain, for the first time, a phenomenon in which the release rate of curcumin varies depending on the film type.

### 3.2. PALS Results

**Figure 3:**
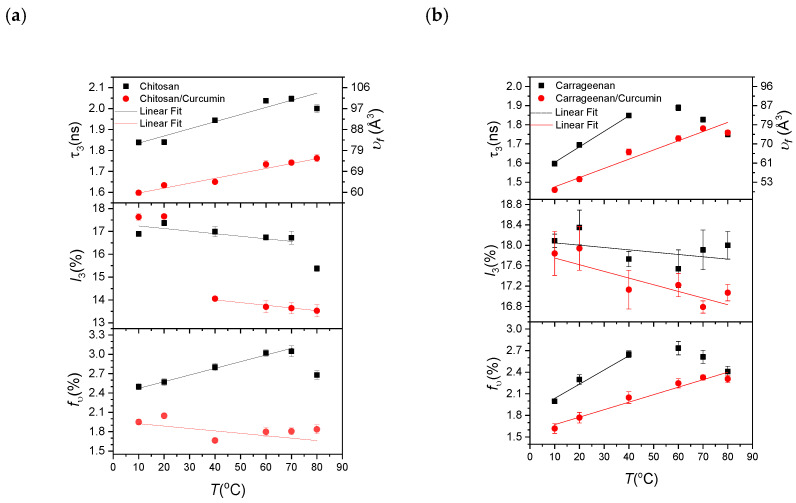
The o-Ps lifetime (with the corresponding free volume hole size on the left axis), intensity, and free volume fraction versus temperature: (**a**) chitosan and chitosan/curcumin sheets, and (**b**) carrageenan and carrageenan/curcumin. Data visibly out of linearity were excluded from the fit.

**Figure 4:**
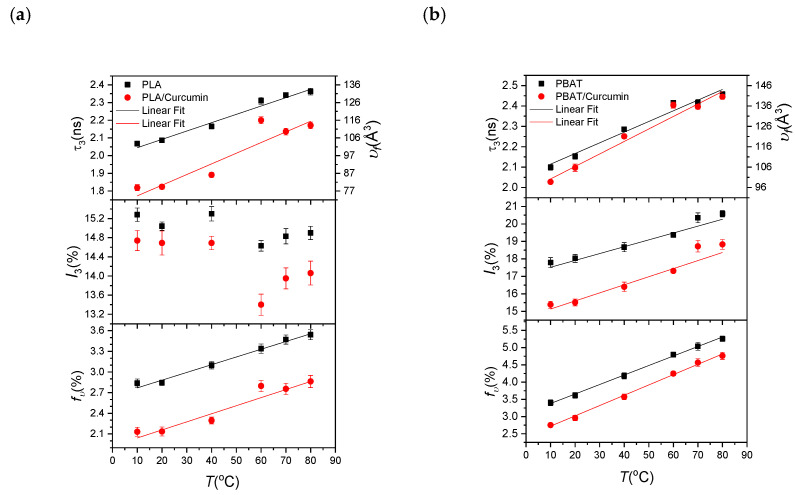
The o-Ps lifetime (with the corresponding free volume hole size on the left axis), intensity, and free volume fraction versus temperature: (**a**) PLA and PLA/curcumin, and (**b**) PBAT and PBAT/curcumin. The solid line is the best fit through the data.

When analyzing the PALS spectrum, we fixed the p-Ps lifetime as τ_1_ = 125 ps, assuming that it is not to change in the vacuum and matter. All the other PALS parameters such as the direct annihilation lifetime (τ_2_) and intensity (*I*_2_), and the o-Ps lifetime (τ_3_) and intensity (*I*_3_) are presented in Table 1. For the chitosan- and carrageenan-based sheets, together with those to which curcumin had been added, parentheses are used, and Table 2 shows the PLA- and PBAT-based sheets together with their curcumin-added sheets in parentheses. In addition, we plotted these data to show the changes in the o-Ps lifetime (left vertical axis) with the resultant free volume hole size (right vertical axis), the o-Ps intensity (*I*_3_), and the free volume fraction (*f_υ_*) for temperatures from 10 to 80 °C in Figure 3 for chitosan and carrageenan, and in Figure 4 for PLA and PBAT. Data visibly out of linearity were excluded from the fit.

#### 3.2.1. Chitosan- and Carrageenan-Based Films as Natural Biopolymers

For the chitosan and carrageenan sheets as natural biopolymers with their curcumin contents, as shown in Figure 3, the o-Ps lifetime (or the free volume hole size) increases almost linearly with the temperature. Still, the o-Ps intensity, as the degree of the hole density, decreases with the temperature. The o-Ps lifetime for the carrageenan and carrageenan/curcumin sheets has a higher slope than the chitosan and chitosan/curcumin sheets, which implies that the carrageenan sheets have a higher thermal expansion coefficient than the chitosan sheets. This behavior is the same for the free volume fraction, as seen in Figure 3. Therefore, the carrageenan sheets have a greater tendency to expand than the chitosan sheets.

On the other hand, the chitosan and carrageenan sheets with curcumin contents have a lower o-Ps lifetime (the free volume hole size) and intensity than their neat sheets. For instance, at 20 °C, the free volume hole size (the free volume fraction) decreases by about 21.6% (20.6%) from 82.2 Å^3^ (2.57%) for chitosan to 64.4 Å^3^ (2.04%) for chitosan/curcumin, and by approximately 21.2% (22.7%) from 69.5 Å^3^ (2.29%) for carrageenan to 54.8 Å^3^ (1.77%) for carrageenan/curcumin. This can be interpreted as the curcumin additive being more eligible to move in the chitosan and carrageenan structure to occupy the free volumes to reduce the free volume hole size and the free volume fraction. In other words, the chitosan and carrageenan structures are rearranged with curcumin to reduce the free volume. However, the thermal slopes of the free volume hole size (in units of Å^3^/°C) have lower values of 0.202 ± 0.020 and 0.443 ± 0.028 for chitosan/curcumin and carrageenan/curcumin, respectively, than chitosan’s and carrageenan’s respective thermal slope values of 0.355 ± 0.035 and 0.716 ± 0.036, respectively. In other words, the chitosan/curcumin and carrageenan/curcumin sheets have a slower increase in the free volume hole size than the chitosan and carrageenan sheets with increasing temperatures. The curcumin content and size had the effect of reducing the free volume hole size; however, its temperature dependence was slightly slower than that of the neat sheets. In other words, it can be said that curcumin has a slight effect on reducing the thermal expansion of the structure. The free volume fraction has a behavior similar to that of the free volume hole size. The thermal expansion coefficient of the free volume fraction in carrageenan/curcumin was lower than that of carrageenan.

As a note, the o-Ps intensity for the chitosan/curcumin sheet has a sharp drop from 40 °C. The pore structures of the chitosan sheet can explain this sharp drop. After 40 °C, the curcumin molecules likely gain some mobility to occupy some part of the pore structures to drop the number of holes in the structure. In addition, there are appreciable reductions in the free volume hole size and free volume fraction of the chitosan sheets after 70 °C and the carrageenan sheets after 60 °C. These can be interpreted as molecular collapse at these high temperatures.

#### 3.2.2. PLA- and PBAT-Based Films as Bioplastic Polymers

For the PLA- and PBAT-based films with their curcumin contents, the o-Ps lifetime with the corresponding free volume hole size and free volume fraction increased almost linearly with the temperature, as shown in Figure 4. On the other hand, the o-Ps intensities of PLA and PLA/curcumin remained virtually constant from 10 to 40 °C; they dropped sharply at 60 °C and increased slightly with the temperature. However, the o-Ps intensities of both PBAT and PBAT/curcumin increased linearly with the temperature. The o-Ps intensities of PLA and PBAT were higher than those of PLA/curcumin and PBAT/curcumin.

PLA/curcumin showed a lower o-Ps lifetime (or free volume hole size) than PLA, with 1.83 ns (80.8 Å^3^) and 2.09 ns (105 Å^3^) at 20 °C, respectively. Thus, curcumin decreased the lifetime (the free volume hole size) by about 12% (23%). On the other hand, the PBAT/curcumin sheet similarly showed a lower o-Ps lifetime (or free volume hole size) than the PBAT sheet, with 2.10 ns (106 Å^3^) and 2.15 ns (111 Å^3^) at 20 °C, respectively. Thus, curcumin slightly decreased the lifetime (the free volume hole size) by about 2.3% (4.5%). However, the thermal slopes of the free volume hole size (in units of Å^3^/°C) have higher values of 0.560 ± 0.111 and 0.612 ± 0.048 for PLA/curcumin and PBAT/curcumin, respectively, than those of PLA and PBAT, with 0.457 ± 0.034 and 0.539 ± 0.043, respectively. In other words, the PLA/curcumin and PBAT/curcumin sheets have a higher increase in the free volume hole size than the PLA and PBAT sheets with increasing temperatures.

The free volume fraction of the PLA and PBAT sheets and their curcumin contents exhibited similar behavior, as shown in Figure 4. Both display a behavior that increases linearly with increasing temperature and are almost parallel to each other. The PLA/curcumin and PBAT/curcumin sheets had 25% and 18% lower free volume fractions than the PLA and PBAT sheets, respectively (e.g., 2.14% and 2.85% for PLA/curcumin and PLA, and 2.96% and 3.61% for PBAT/curcumin and PBAT, respectively, at 20 °C). Curcumin causes a greater free volume reduction in PLA than in PBAT.

From the PALS results of curcumin when it was incorporated in various types of polymer-based films, it can be seen that the release of curcumin relies on the polymer film’s free volume. Figure 1 and Figure 2 show that curcumin was released most rapidly in the carrageenan film, followed by the chitosan, PBAT, and PLA films. The free volume hole size was reduced when curcumin was added to the polymer matrix. The curcumin-added carrageenan film had the lowest free volume hole size, followed by chitosan, PLA, and PBAT, while PLA and PBAT had similar free volume hole sizes (o-Ps lifetime). A reduction in the free volume hole size decreases the void space of the polymer chains, leading to reorganization, impeding chain mobility, and reducing viscosity while enhancing the elastic behavior of the material [36]. The blending of curcumin condensed the free volume fraction of all the polymer matrices.

Furthermore, the decline in the free volume fraction by the addition of curcumin was smaller in the natural biopolymer-based films compared to the bioplastic-based films. The free volume fraction of the curcumin-incorporated films was also lower in the order of carrageenan (1.7%), chitosan (2.05%), PLA (2.14%), and PBAT (2.96%). It has been reported that the addition of fillers in a polymer matrix reduces the fraction of the free volume in the polymer matrix [37]; however, the effect of the interface of fillers on the free volume of polymer composites is not yet fully understood, although a recent publication studied these effects for PLA with cellulose nanoparticles [38].

Based on the free volume analysis of the curcumin-added polymers, the free volume hole size and free volume fraction of the carrageenan-based film were the lowest, showing the fastest and greatest curcumin release. In contrast, curcumin release was much slower in the bioplastic-based films, presumably due to the higher free volume hole size and free volume fraction. However, the release of curcumin in the PLA- and PBAT-based films was very similar due to the similar free volume hole size in the two polymers. This study is a preliminary investigation to explore the correlation between the free volume and the release of bioactive compounds in various polymer complexes. Further work is needed to better understand the correlation between these two parameters.

## 4. Conclusions

The release rate of curcumin from various polymer (carrageenan, chitosan, PLA, and PBAT) films was evaluated and associated with the phenomenon of free volume measured by PALS analysis. The release of curcumin in aqueous media mainly depended on the type of polymer matrix film. The free volume of various matrix polymer films and curcumin-added composite films also relied on the type of matrix polymer. Despite the simplification, the free volume method has been established to provide an effective correlation and anti-prediction tool for releasing bioactive compounds from functional films. Current research proposes that the polymer matrix’s free volume has a substantial influence on the release of curcumin in aqueous media. This phenomenon could be fundamental to fully comprehending the release of bioactive compounds in polymer-based composite films.

## Figures and Tables

**Table 1:** PALS parameters as lifetimes (τ_2_ and τ_3_) and intensities (*I*_2_ and *I*_3_) with free volume hole size (*υ_f_*) and free volume fraction (*f_υ_*) in terms of temperature for chitosan, chitosan/curcumin, carrageenan, and carrageenan/curcumin.

Films	T (°C)	τ_2_ (ns) (±0.003)	*I*_2_ (%) (±0.8)	τ_3_ (ns) (±0.01)	*I*_3_ (%) (±0.3)	*υ_f_* (Å^3^) (±1.4)	*f_υ_* (%) (±0.11)
Chitosan (chitosan/curcumin)	10	0.378 (0.358)	68.4 (77.0)	1.84 (1.60)	16.9 (17.6)	82.1 (61.4)	2.50 (1.95)
	20	0.372 (0.352)	70.9 (79.9)	1.84 (1.63)	17.4 (17.7)	82.2 (64.4)	2.57 (2.05)
	40	0.368 (0.336)	72.5 (70.0)	1.94 (1.65)	17.0 (14.1)	91.6 (65.7)	2.80 (1.66)
	60	0.372 (0.343)	70.8 (66.1)	2.05 (1.73)	16.7 (13.7)	100 (72.6)	3.02 (1.80)
	70	0.361 (0.341)	76.1 (67.8)	2.05 (1.74)	16.7 (13.6)	101 (73.6)	3.05 (1.81)
	80	0.375 (0.342)	69.6 (68.2)	2.00 (1.76)	15.4 (13.5)	96.8 (75.4)	2.68 (1.84)
Carrageenan (carrageenan/curcumin)	10	0.359 (0.352)	75.9 (75.9)	1.60 (1.46)	18.1 (17.8)	61.3 (50.4)	2.00 (1.62)
	20	0.354 (0.352)	76.7 (76.5)	1.70 (1.52)	18.4 (17.9)	69.5 (54.8)	2.30 (1.77)
	40	0.358 (0.361)	76.8 (74.9)	1.85 (1.66)	17.7 (17.1)	83.0 (66.4)	2.65 (2.05)
	60	0.361 (0.357)	76.7 (76.7)	1.89 (1.73)	17.5 (17.2)	86.6 (72.6)	2.73 (2.25)
	70	0.358 (0.366)	74.7 (73.6)	1.83 (1.78)	17.9 (16.8)	81.0 (77.0)	2.61 (2.33)
	80	0.352 (0.361)	77.7 (75.0)	1.75 (1.76)	18.0 (17.1)	74.4 (75.1)	2.41 (2.31)

The intensities were normalized as *I*_1_ + *I*_2_ + *I*_3_ = 1.

**Table 2:** PALS parameters as lifetimes (τ_2_ and τ_3_) and intensities (*I*_2_ and *I*_3_) with free volume hole size (*υ_f_*) and free volume fraction (*f_υ_*) in terms of temperature for PLA, PLA/curcumin, PBAT, and PBAT/curcumin.

Films	T (°C)	τ_2_ (ns) (±0.003)	*I*_2_ (%) (±0.8)	τ_3_ (ns) (±0.01)	*I*_3_ (%) (±0.3)	*υ_f_* (Å^3^) (±1.6)	*f_υ_* (%) (±0.11)
PLA (PLA/curcumin)	10	0.382 (0.376)	75.7 (71.8)	2.07 (1.82)	15.3 (14.7)	103 (80.3)	2.84 (2.13)
	20	0.384 (0.379)	74.6 (71.5)	2.09 (1.83)	15.0 (14.7)	105 (80.8)	2.84 (2.14)
	40	0.374 (0.379)	80.0 (73.1)	2.17 (1.89)	15.3 (14.7)	113 (86.8)	3.10 (2.29)
	60	0.378 (0.402)	78.4 (61.9)	2.31 (2.20)	14.6 (13.4)	127 (116)	3.34 (2.80)
	70	0.380 (0.379)	77.2 (72.9)	2.34 (2.14)	14.8 (14.0)	130 (110)	3.47 (2.76)
	80	0.382 (0.381)	76.2 (72.1)	2.36 (2.17)	14.9 (14.1)	132 (113)	3.54 (2.86)
PBAT (PBAT/curcumin)	10	0.357 (0.346)	67.1 (70.7)	2.10 (2.03)	17.8 (15.4)	106 (99.4)	3.40 (2.75)
	20	0.365 (0.350)	63.2 (69.5)	2.15 (2.10)	18.0 (15.5)	111 (106)	3.61 (2.96)
	40	0.362 (0.360)	65.7 (66.8)	2.29 (2.25)	18.7 (16.4)	124 (121)	4.18 (3.57)
	60	0.361 (0.360)	65.6 (69.5)	2.42 (2.40)	19.4 (17.3)	138 (136)	4.80 (4.25)
	70	0.360 (0.351)	64.8 (68.7)	2.42 (2.40)	20.4 (18.7)	138 (136)	5.04 (4.57)
	80	0.360 (0.367)	64.9 (61.5)	2.46 (2.45)	20.6 (18.8)	142 (141)	5.25 (4.76)

The intensities were normalized as *I*_1_ + *I*_2_ + *I*_3_ = 1.

## Data Availability

Data available on request due to restrictions e.g., privacy or ethical.
